# More is More? Total Pancreatectomy for Periampullary Cancer as an Alternative in Patients with High-Risk Pancreatic Anastomosis: A Propensity Score-Matched Analysis

**DOI:** 10.1245/s10434-021-10292-8

**Published:** 2021-06-24

**Authors:** Sebastian Hempel, Florian Oehme, Ermal Tahirukaj, Fiona R. Kolbinger, Benjamin Müssle, Thilo Welsch, Jürgen Weitz, Marius Distler

**Affiliations:** Department of Visceral, Thoracic and Vascular Surgery, University Hospital and Faculty of Medicine Carl Gustav Carus, Technische Universität Dresden, Dresden, Germany

## Abstract

**Background:**

Postpancreatectomy morbidity remains significant even in high-volume centers and frequently results in delay or suspension of indicated adjuvant oncological treatment. This study investigated the short-term and long-term outcome after primary total pancreatectomy (PTP) and pylorus-preserving pancreaticoduodenectomy (PPPD) or Whipple procedure, with a special focus on administration of adjuvant therapy and oncological survival.

**Methods:**

Patients who underwent PTP or PPPD/Whipple for periampullary cancer between January 2008 and December 2017 were retrospectively analyzed. Propensity score-matched analysis was performed to compare perioperative and oncological outcomes. Correspondingly, cases of rescue completion pancreatectomy (RCP) were analyzed.

**Results:**

In total, 41 PTP and 343 PPPD/Whipple procedures were performed for periampullary cancer. After propensity score matching, morbidity (Clavien-Dindo classification (CDC) ≥ IIIa, 31.7% vs. 24.4%; *p *= 0.62) and mortality rates (7.3% vs. 2.4%, *p *= 0.36) were similar in PTP and PPPD/Whipple. Frequency of adjuvant treatment administration (76.5% vs. 78.4%; *p *= 0.87), overall survival (513 vs. 652 days; *p *= 0.47), and progression-free survival (456 vs. 454 days; *p *= 0.95) did not significantly differ. In turn, after RCP, morbidity (CDC ≥ IIIa, 85%) and mortality (40%) were high, and overall survival was poor (median 104 days). Indicated adjuvant therapy was not administered in 77%.

**Conclusions:**

In periampullary cancers, PTP may provide surgical and oncological treatment outcomes comparable with pancreatic head resections and might save patients from RCP. Especially in selected cases with high-risk pancreatic anastomosis or preoperatively impaired glucose tolerance, PTP may provide a safe treatment alternative to pancreatic head resection.

**Supplementary Information:**

The online version contains supplementary material available at 10.1245/s10434-021-10292-8.

Complete tumor resection remains an obligatory part of curative treatment strategies for periampullary malignancies. The surgical standard approach is pancreaticoduodenectomy (PPPD/Whipple). Pancreatic head resections, however, are associated with significant perioperative morbidity and mortality, even in high-volume centers. According to Nimptsch et al., the in-hospital mortality rate after PPPD and Whipple procedure averages 10% in Germany.^1^ Postoperative pancreatic fistula (POPF) results from leakage or insufficiency of the pancreatoenteric anastomosis and is one of the most dreaded complications of partial pancreatic resection, affecting up to 40% of patients.^2^ While clinically relevant POPFs can be life-threatening through single or multiple organ failure themselves, POPF also is a driver for further potentially lethal complications, such as postpancreatectomy hemorrhage (PPH). POPF and POPF-associated complications both extend hospital stay and frequently result in long-term decline of patients’ general health condition. In patients with malignant diseases, a complicated clinical course can be particularly relevant through a delay of adjuvant oncological therapy, thus directly impacting oncological outcome.

While PPPD and Whipple procedure are the most commonly performed pancreatic resections, primary total pancreatectomy (PTP) is indicated in selected patients with extended chronic pancreatitis, neoplasms involving the entire pancreas or locally advanced pancreatic tumors. Initially, PTP was proposed to both avoid postoperative complications associated with anastomotic leakage and prevent disease recurrence through clinically inapparent synchronous disease in the gland.^3^ Given its inherent consequence of permanent exocrine and endocrine insufficiency, PTP was poorly accepted by most surgeons in the past,^4^ and adverse effects on the quality of life and long-term outcome of patients have been described in former decades.^5^,^6^

In recent years, management and treatment of both endocrine and exocrine pancreatic insufficiency have been optimized. The introduction of novel insulin regimens and delivery devices has led to improved treatment of insulin-dependent diabetes mellitus (IDDM).^7^ In patients with exocrine insufficiency of the pancreas, improved pancreatic enzyme supplementation and antihypertensive medication have contributed markedly to improved quality of life.^8^

To date, these relevant therapeutic improvements have, however, not been reflected in surgical guidelines. Therefore, we reevaluated the surgical outcome of PTP versus standard pancreatic head resection in patients with periampullary cancers and evaluated postoperative morbidity and oncological outcome in patients undergoing PTP and PPPD/Whipple with a special focus on adjuvant treatment. Last, we analyzed the outcomes of all patients who underwent RCP secondary to pancreatic head resection for periampullary cancer within the study period.

This study primarily compared oncological overall und progression-free survival for patients who underwent PPPD/Whipple and PTP. Based on these results, a second goal was to provide a basis for evidence-based discussion of total pancreatectomy as a surgical treatment approach in patients with periampullary malignancies to define individualized surgical treatment approaches that consider patients’ varying risks of complications following partial pancreatic resection.

## Methods

### Study Design and Patient Data Acquisition

**Fig. 1:**
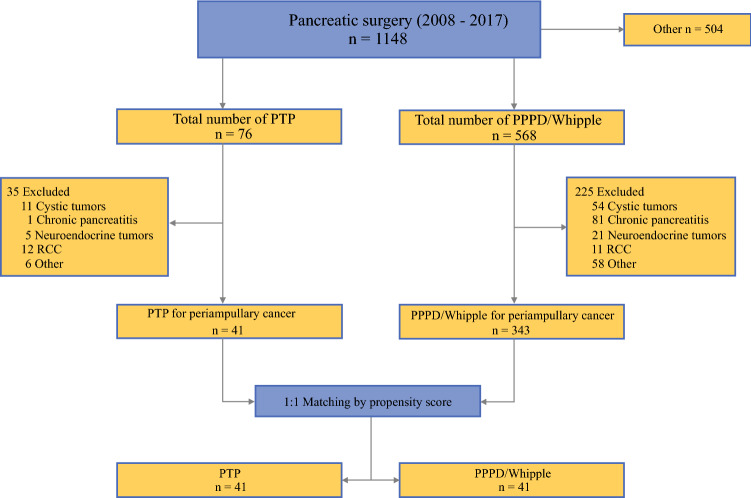
Cases included in and excluded from the study. *PPPD* pylorus-preserving pancreaticoduodenectomy; *PTP* primary total pancreatectomy; *RCC* renal cell carcinoma metastases

This study was designed as a retrospective observational study. All patients who underwent pancreatic head resection (PPPD/Whipple) and PTP for periampullary cancer between January 2008 and December 2017 at the Department of Visceral, Thoracic and Vascular Surgery, University Hospital Carl Gustav Carus, Technische Universität Dresden, Germany, were included in this study and subsequently subjected a 1:1 propensity score-matched analysis. A corresponding flow-chart depicting all cases in and excluded in the process is shown in Fig. 1.

Medical records, including overall survival (OS) and progression-free survival (PFS) for each case, were obtained from a prospective database and retrospectively analyzed. Furthermore, data on administration and suspension of indicated adjuvant treatment following PTP and PPPD/Whipple were collected from the local clinical information and tumor documentation system. Fistula risk score (FRS),^9^ alternative FRS (aFRS),^10^ and updated alternative FRS (ua-FRS)^11^ were calculated based on preoperative and intraoperative data. In cases of PTP, the pancreatic duct diameter was determined based on preoperative imaging.

Follow-up data on tumor recurrence was obtained during regular examinations in our outpatient clinic as well as through phone calls or interviews with primary care physicians. Tumor progression was detected by case history and clinical examination, an elevated carbohydrate antigen 19-9 (CA 19-9) level or radiographically detected tumor recurrence (e.g., sonography, computed tomography (CT), or magnetic resonance imaging (MRI) scans).

In addition, outcome data of all patients who underwent RCP during the study period were determined.

The experimental protocol of the study was approved by the local ethics committee of the TU Dresden (decision number EK 310062019). All methods were performed in accordance with relevant guidelines. Informed consent was obtained from all included patients.

### Definitions and Operative Technique

*Primary total pancreatectomy (PTP)* subsumed planned total resection of the pancreas and total resection of the pancreas resulting from an intraoperative decision in order to achieve negative resection margins or circumvent POPF-associated complications.

*Rescue completion pancreatectomy (RCP)* was defined as surgical removal of all pancreatic remnant tissue in a secondary surgical procedure after initial partial pancreatic resection. RCP was indicated in cases of major complications after pancreatic head resections that could not be managed conservatively.

All PTP and PPPD/Whipple procedures were performed as elective, open surgeries.

### Outcomes

The primary endpoint was oncological outcome, measured in median overall survival, median progression-free survival, and completion of indicated adjuvant therapy. The secondary endpoints included intraoperative measurements (duration of surgery, intraoperative blood loss, fistula risk scores), indicators of the postoperative course (median hospital and ICU stay, postoperative ECOG performance status), and the overall morbidity and mortality, including postoperative complications according to the Clavien-Dindo classification. Last, potential risk factors of a situation necessitating RCP were determined through the analysis of all patients undergoing RCP during the study period to identify patients who could benefit from PTP.

### Statistical Analysis

For comparison of categorical and quantitative variables, Fisher’s exact test and two-tailed unpaired *t*-test (normally distributed data) or a Mann-Whitney *U* test (not normally distributed data) were used, respectively. *p *< 0.05 was considered statistically significant. Categorical data were expressed as patient number and percentage of the respective patient cohort. Quantitative variables were reported as median and interquartile range (IQR).

We performed a propensity score matched analysis between PPPD and PTP using the nearest neighbor method to 1:1 ratio. Propensity score deviation width was set to a threshold of < 0.3. Variables used for matching were age, sex, and prognostically relevant factors: tumor size, nodal status and resection status. To detect residual imbalances after matching we employed a standardized mean deviation analysis.

Using the Kaplan–Meier method, the overall survival (OS) and progression-free survival (PFS) curves were calculated. Log-rank test served to compare OS and PFS between these groups. OS was defined as the time interval between the index surgery (PPPD/Whipple or PTP) and the date of death or time of last contact (censored). Accordingly, PFS was defined as the time between the index surgery and the last follow-up without tumor progression. The period from surgery to last patient contact or death of the patient was defined as follow-up time.

A competitive analysis considering body mass index (BMI), FRS^9^, aFRS^10^, ua-FRS^11^, serum amylase levels on POD 1 and 2, intraoperative blood loss, multivisceral resection, and operation time was performed analyzing differences between PPPD/Whipple, PTP, and RCP regarding potential risk factors for the development of a RCP situation. For data visualization, the IBM SPSS 25 (SPSS Statistics v25, IBM Corporation, Armonk, NY) software package and Microsoft Office 2019 (Microsoft Corporation, Redmond, WA) were used.

## Results

### Characteristics of Propensity Score-Matched Patients Undergoing PPPD/Whipple and PTP

Of 343 PPPD/Whipple procedures performed during the study period, a 1:1 propensity score matching method was used to determine and compare 41 pairs of PTP and PPPD/Whipple procedure cases according to the abovementioned oncological outcome-related and histopathological variables (Table 1). The two groups showed small differences with regard to effect sizes of the matched variables age (SMD 0.12), sex (SMD 0.05), tumor size (SMD 0.17), N-status (SMD 0.25), and R-status (SMD 0.11). Furthermore, the parameters ASA score II (SMD 0.16) and III (SMD 0.14), preoperative weight loss (SMD 0.1), and comorbidities, including diabetes (SMD 0.25), alcohol abuse (SMD 0.06), nicotine abuse (SMD 0.2), and hypertension (SMD 0.15), did not differ significantly between the groups. PPPD/Whipple patients displayed a trend toward higher preoperative CA 19-9 levels (median 129.4 U/l vs. 42.8 U/l), although the SMD of 0.16 was small.

**Table 1:** Characteristics of patients undergoing PPPD/Whipple and PTP

Variable	PPPD/Whipple	PTP	SMD
Patients (*n*)	41	41	
Median age (years) (IQR)	67 (63–72)	67 (61–71)	0.12
Male sex (*n* (%)]	20 (48.8)	19 (46.3)	0.05
ASA Score [*n* (%)]			
1	8 (19.5)	2 (4.9)	0.46
2	19 (46.4)	22 (53.7)	0.16
3	14 (34.1)	17 (41.4)	0.14
Diabetes [*n* (%)]	20 (48.8)	15 (36.6)	0.25
Weight loss [*n* (%)]	26 (63.4)	28 (68.3)	0.1
Jaundice [*n* (%)]	30 (73.2)	20 (48.8)	0.52
Alcohol abuse [*n* (%)]	7 (17.1)	8 (19.5)	0.06
Nicotine abuse [*n* (%)]	5 (12.2)	8 (19.5)	0.2
Hypertension [*n* (%)]	27 (65.9)	24 (58.5)	0.15
Median CA 19–9 [U/ml] (IQR)	129.4 (55.6–550)	42.8 (14–163)	0.16
Neoadjuvant therapy [*n* (%)]	1 (2.4)	12 (29.3)	0.79
Portal vein resection [*n* (%)]	12 (29.3)	23 (56.1)	0.56
Arterial resection [*n* (%)]	1 (2.4)	19 (46.3)	1.19
Histology [*n* (%)]			0.46
PDAC	41 (100)	37 (90.2)	
Distal bile duct cancer	–	4 (9.8)	
Ampullary cancer	–	–	
Tumor size [mm]	31.5 (25–39.3)	32.5 (21.5–49.3)	0.17
N			
N0	15 (36.6)	20 (48.8)	0.25
N1	20 (48.8)	15 (36.6)	
N2	6 (14.6)	6 (14.6)	
R			0.11
R0	31 (75.6)	29 (70.7)	
R1	10 (24.4)	12 (29.3)	

*ASA* American Society of Anesthesiologists; *CA19-9* carbohydrate antigen 19-9; *IQR* interquartile range; *PDAC* pancreatic ductal adenocarcinoma; *PTP* primary total pancreatectomy; *SMD* standard mean difference

Despite accurate propensity score matching, patients in the PTP group tended to suffer from more advanced tumors than patients in the PPPD/Whipple group. Consequently, patients who underwent PTP received neoadjuvant therapy more often (29.3% vs. 2.4%; SMD 0.79) than patients who underwent standard pancreatic head resection. In addition, more concomitant arterial (46.3% vs. 2.4%; SMD 1.19) or portal vein resections (56.1% vs. 29.3%; SMD 0.56) were performed during PTP.

### Perioperative Outcomes

Compared with patients undergoing PTP, PPPD/Whipple patients had a significantly shorter length of hospital stay (15 vs. 21 days; *p *< 0.01) and ICU treatment period (4 vs. 7 days; *p *= 0.08). Although patients in PTP underwent more extended resection, there were no significant differences in postoperative morbidity and mortality between the PTP and PPPD/Whipple group. Postoperative morbidity was comparable at an overall occurrence of complications in 75.6% of patients undergoing PPPD/Whipple and 82.9% of patients undergoing PTP (*p *= 0.58). In particular, there was no significant difference regarding the number of severe complications (CDC ≥ IIIa) (PPPD/Whipple: 24.4% vs. PTP: 31.7%; *p *= 0.62). The number of reoperations (4 vs. 8, *p *= 0.35) and in-hospital mortality rate (2.4% vs. 7.3%, *p *= 0.36) did not significantly differ between the two groups. Reasons for reoperation were ischemia (PTP: 3 vs. PPPD/Whipple: 1); bile leakage (PTP: 1 vs. PPPD/Whipple: 1); intra-abdominal hematoma (PTP: 2 vs. PPPD/Whipple: 1); and burst abdomen (PTP: 2 vs. PPPD/Whipple: 1). ECOG performance status 12 months after surgery (*p *= 0.97) and median HbA1c levels (*p *= 0.08) did not differ significantly (Table 2).

**Table 2:** Short- and long-term outcome after PTP and PPPD/Whipple for periampullary cancer

Variable	PPPD/Whipple (*n *= 41)	PTP (*n* = 41)	*P *value
Length of hospital stay (days) (IQR)	15 (13–19)	21 (16.5–30.5)	**<0.01**
Length of ICU stay (days) (IQR)	4 (3–5)	7 (6–10)	0.08
In-hospital mortality [*n* (%)]	1 (2.4)	3 (7.3)	0.36
Overall complications [*n* (%)]	31 (75.6)	34 (82.9)	0.58
CDC ≥ IIIa	10 (24.4)	13 (31.7)	0.62
Reoperation [*n* (%)]	4 (9.8)	8 (19.5)	0.35
Adjuvant therapy [*n* (%)]			0.87
Indicated	37 (100)	34 (100)	
Received	29 (78.4)	26 (76.5)	
Overall survival (days) (CI)	652 (516–787)	513 (281–745)	0.47
Progression-free survival (days) (CI)	454 (280–627)	456 (194–717)	0.95
Follow-up time (days) (IQR)	522 (279–913)	510 (223–672)	0.82
ECOG performance status [*n* (%)]*			0.97
0	19	18	
1–2	20	21	
3–4	2	2	
Median HbA1c (mmol/L) (IQR)*	6.4 (6.1–7.0)	6.7 (6.6–8.3)	0.08

^*^12 months postoperatively

CDC, Clavien-Dindo classification; CI, 95% confidence interval; ECOG, Eastern Cooperative Oncology Group; ICU, intensive care unit; IQR, interquartile range; PPPD, pylorus-preserving pancreaticoduodenectomy; PTP, primary total pancreatectomy

### Oncological Outcomes

**Fig. 2:**
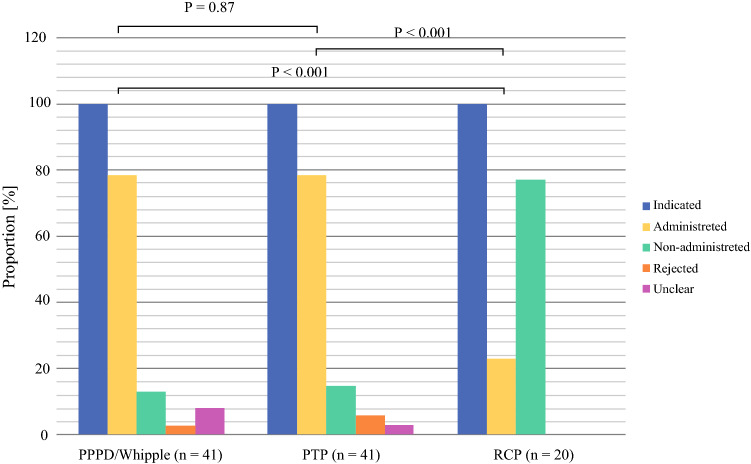
Indication, administration and suspension of adjuvant therapy after PPPD/Whipple PTP and RCP. Number of patients with indicated adjuvant therapy within PPPD/Whipple (*n *= 37), PTP (*n *= 34) and RCP (*n *= 13) were considered as 100%. *PPPD* pylorus-preserving pancreaticoduodenectomy, *PTP* primary total pancreatectomy, *RCP* rescue completion pancreatectomy

After PTP, adjuvant chemotherapy was indicated in 34 cases, and adjuvant treatment was actually administered in 26 patients (76.5%). In the PPPD/Whipple group, 29 of 37 patients (78.4%) indicated cases received adjuvant treatment. Regarding administration of adjuvant treatment, there was no significant difference between both groups (*p *= 0.87). The nonadministration rate for patients with indicated adjuvant treatment was 14.7% in the PTP group and 10.8% in the PPPD/Whipple group. Adjuvant treatment was rejected by two PTP patients and one patient in the PPPD/Whipple group (Fig. 2).

**Fig. 3:**
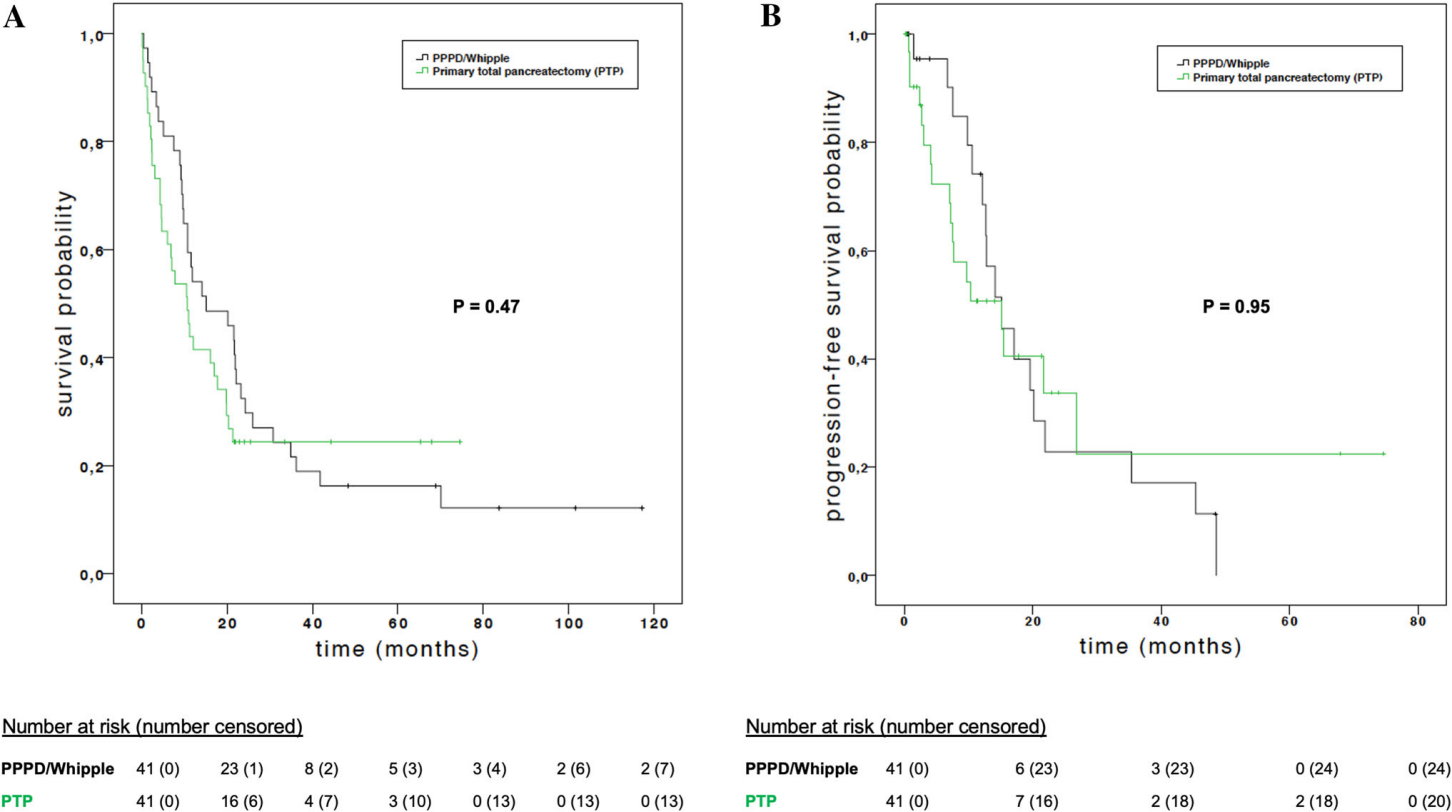
Overall survival and progression-free survival of propensity score-matched patients after PTP (*n *= 41) and PPPD/Whipple (*n *= 41). Overall survival curves (**a**) and progression-free survival (**b**) were plotted

Figure 3 illustrates overall and progression-free survival of PTP patients and matched PPPD/Whipple patients. Patients who underwent PTP had an OS of 513 days (95% confidence interval [CI] 281–745) compared with 652 days for the PPPD/Whipple group (95% CI 516–787); there was no significant difference (*p *= 0.47). Likewise, no difference was seen between both groups with regard to PFS (*p *= 0.95). In the PTP group, PFS was 456 days (95% CI 194–717) versus 454 days in the PPPD/Whipple group (95% CI 280–627). Causes of death within the follow-up period were cancer (PTP: 23 vs. PPPD/Whipple: 31), multiple organ failure (MOF) as a complication of surgery (PTP: 3 vs. PPPD/Whipple: 1), and two cases of acute myocardial infarction in the PTP group. Contrarily, patients who underwent RCP had significantly worse outcomes (Supplementary Table 1).

### Potential Risk Factors for the Development of an RCP Indication after Pancreatic Head Resection

In addition, we assessed potential risk factors for the development of a RCP indication by analyzing all patients undergoing RCP during the study period (Table 3). No significant differences between PPPD/Whipple, PTP and RCP patients were observed with regard to BMI (*p *= 0.11), intraoperative blood loss (*p *= 0.48), and operation time (*p *= 0.33). In contrast, FRS, aFRS, ua-FRS, and the proportion of patients with elevated serum amylase on POD 1 and 2 differed significantly. In the RCP group, the median FRS according to Callery et al., was significantly higher (7, interquartile range [IQR] 5–7) than in the PPPD/Whipple (3, IQR 2–4, *p *< 0.001) or PTP group (4, IQR 3–5, *p *< 0.01). In the PPPD/Whipple and PTP group, the median aFRS according to Mungroop et al. was similar (6% vs. 8%) and classified as intermediate risk. In turn, the median aFRS in the RCP group was 20.2% and differed significantly from the aforementioned groups (*p *< 0.001). The median ua-FRS also was significantly higher in the RCP group (53, IQR 34–60) than in the PPPD/Whipple (14, IQR 12–23, *p *< 0.001) and the PTP groups (18, IQR 13–29, *p *< 0.001). Altogether, the three fistula risk scores indicated a high-risk constellation of POPF development in the RCP group. Furthermore, significantly elevated serum amylase levels were observed in the RCP group compared with the PPPD/Whipple and PTP groups on POD 1 (137.4, IQR 83.4–195, U/l, *p *< 0.01) and POD 2 (61.8, IQR 29.4–129, U/l, *p *< 0.001).

**Table 3:** Potential risk factors for the development of a RCP situation

Variable	PPPD/Whipple	PTP	RCP^a^	*p* value
BMI	25.1 (22.7–28)	24.3 (22.2–26.3)	27.1 (24.2–30.2)	0.11
Median FRS^9^	3 (2–4)	4 (3–5)	7 (5–7)	< **0.001**^**b**^
Median aFRS^10^ (%) (IQR)	6 (3.1–8.2)	8 (4.8–13)	20.2 (12.3–28.7)	< **0.001**^**c**^
Median ua-FRS^11^ (%) (IQR)	14 (12–23)	18 (13–29)	53 (34–60)	< **0.001**^**c**^
Serum amylase (U/l) (IQR)				
POD 1	28.8 (10.8–76.2)	N.A.	137.4 (83.4–195)	< **0.01**
POD 2	11.4 (6.6–36.6)	N.A.	61.8 (29.4–129)	< **0.001**
Intraoperative blood loss (ml) (IQR)	1000 (600–1500)	1000 (500–1700)	900 (513–1150)	0.48
Multivisceral resection (*n*/%)	3 (7.3)	31 (75.6)	2 (10)	< **0.001**
Operation time (min) (IQR)	419 (340–519)	457 (362–545)	424 (297–593)	0.33

*BMI* body mass index; *FRS* fistula risk score; *aFRS* alternative fistula risk score; *N.A.* not available; *POD* postoperative day; *PTP* primary total pancreatectomy; *RCP* rescue completion pancreatectomy; *ua-FRS* updated alternative fistula risk score

^a^All RCP cases for periampullary cancer within the study period (unmatched)

^b^PPPD versus RCP: < 0.001; PTP versus RCP: < 0.01

^c^PPPD versus RCP: < 0.001; PTP versus RCP: < 0.001

## Discussion

Over the past decades, perioperative morbidity and mortality after pancreatic surgery have improved due to several factors, including better surgical techniques, earlier detection and management of complications, and centralization of pancreatic surgery in high-volume centers.^12^–^14^ Still, postpancreatectomy complications, such as POPF and POPF-associated hemorrhage affect up to 40% of patients and are associated with poor short-term and oncological outcome. In severe cases of anastomosis-related complications, in which the situs is not suitable for anastomosis repair (e.g. due to remnant pancreatitis), RCP serves as ultima ratio therapeutic option, which, in line with our findings, is associated with high in-hospital mortality rates between 24 and 64%.^15^–^19^ Moreover, RCP has been associated with longer in-hospital stay (median ranging from 34 to 55 days)^15^,^16^,^20^,^21^ and deterioration of the patient’s general health.

While PTP avoids postoperative complications resulting from pancreatoenteric anastomotic leakage or insufficiency, it results in total exocrine and endocrine pancreatic insufficiency—two clinical challenges that necessitate close monitoring and life-long treatment. While high overall morbidity rates of 40–70% have been reported for TP in the past,^22^–^25^ data on the impact of PTP on quality of life is heterogeneous, particularly due a lack of discrimination between PTP and RCP in most publications. While some studies^23^,^26^–^30^ have described a reduction in general health perception and physical status, other groups have reported no significant differences after total and partial pancreatectomy in a large patient cohort.^23^ Pulvirenti et al. concluded that older patients reported better quality of life than younger patients.^28^ In the context of a median overall survival of approximately 17 months, the impact of IDDM and its secondary complications on quality of life after oncological pancreatic resection also may be less pronounced than in health conditions with a better overall prognosis. Future in-depth evaluations of the impact of surgical treatment approaches for pancreatic malignancies on quality of life should consider advances in both pancreatic surgery (e.g., concomitant islet cell autotransplantation) and management of pancreatic insufficiency (e.g., modern glucose monitors and insulin delivery devices).

Recent studies have reported that PTP can be performed with acceptable morbidity (approximately 22.5–48.0%) and mortality (approximately 3.3–6.0%),^4^,^8^,^22^,^28^ which is comparable to our findings. While the overall complication rate of PTP was high in our study (83%), most of these complications were minor (CDC I or II, 51%). Similar to our findings, Reddy et al. have reported minor complications in 59% of the cases in their large, single-center experience.^24^ Compared with patients undergoing standard pancreatic head resection, our study identified both hospital and ICU stay were significantly longer in patients undergoing PTP, an explanation for which could be the need for treatment and management of the exocrine and endocrine insufficiency after surgery. In contrast to the findings of Passeri et al.,^4^ similar proportions of patients undergoing PTP and PPPD/Whipple who had an indication for adjuvant therapy received adjuvant treatment. In line with the results for pancreatic adenocarcinoma reported by Reddy et al.,^24^ our study demonstrated equivalent overall survival and progression-free survival following PTP and PPPD/Whipple.

Our findings on the surgical and oncological safety of PTP and the dismal prognosis of RCP imply the necessity to prevent grade C POPF resulting in RCP. In recent years, various risk scores have been developed and validated for risk stratification, taking both preoperatively available and intraoperative data into account. Our data confirm that patients requiring RCP during the clinical course had high fistula risk scores in three of the most well-established scoring systems, the FRS developed by Callery et al.^9^ and the aFRS developed by Mungroop et al.^10^, as well as the updated alternative FRS by Mungroop et al.^11^.

The limitations of this study are first its retrospective character and heterogeneity regarding tumor localization and extension of resection, which is especially reflected in the concomitant arterial reconstructions in the PTP group. Second, given the small group size, the current study is likely underpowered to make definitive claims on outcomes. Still, it provides evidence that PTP and PPPD/Whipple provide comparable oncological outcomes in patients with periampullary cancer. Third, there is a lack of questionnaire-based data regarding quality of life after PTP; however, our study demonstrates comparable postoperative ECOG performance status and HbA1c levels between both groups. Further limitations related to the statistical methodology are firstly due the limited number of known confounders that were used as matching variables, implying that residual confounding is likely. Moreover, the PS match caliper was exceptionally set at < 0.3, because a lower a PS match caliper would have resulted in considerably fewer matching pairs, despite a relatively large control group. Third, we treated the data missing as “missing completely at random,” which may lead to a potential attrition bias.

## Conclusions

This study demonstrates that PTP may provide similar oncological outcomes to standard pancreatic head resection with regard to overall and progression-free survival. In particular, the completion of indicated adjuvant therapy did not significantly differ between patients undergoing PTP and PPPD/Whipple. Because RCP as a last-resort treatment option in patients with severe POPF, in turn, is associated with adverse short-term and long-term outcomes, PTP should be considered a viable treatment option in patients with periampullary cancers who, according to available risk stratification systems, are likely to suffer from POPF. With regard to recent advances in the management of both endocrine and exocrine pancreatic insufficiency, our findings encourage future studies of the quality of life after partial and total pancreatic resection as well as studies of the preoperative identification of patients at high risk of developing postpancreatectomy complications.

## Supplementary Information

Below is the link to the electronic supplementary material.

Supplementary file1 (DOCX 13 kb)
