# Supplementary material for: More is More? Total Pancreatectomy for Periampullary Cancer as an Alternative in Patients with High-Risk Pancreatic Anastomosis: A Propensity Score-Matched Analysis
Source: Ann Surg Oncol. 2021 Jun 24;28(13):8309–17. doi: 10.1245/s10434-021-10292-8 (PMC8590996; doi:10.1245/s10434-021-10292-8)
Supplement: Supplementary file 1 — Supplementary file1 (DOCX 13 kb) [file 10434_2021_10292_MOESM1_ESM.docx]

**Supplementary table.** Short- and long-term outcome after RCP in patients with periampullary cancer.

| **Variable** | **RCP (n=20)** | **P-value^*^** |
| --- | --- | --- |
| Histology [n (%)] |  | **-** |
| *PDAC* | 7 (35.0) |  |
| *Distal bile duct cancer* | 7 (35.0) |  |
| *Ampullary cancer* | 6 (30.0) |  |
| POD of reoperation (IQR) | 10 (6-14) | **-** |
| Preoperative SOFA score (IQR) | 5 (3-10) | **-** |
| Length of hospital stay (LOS) [days] (IQR) | 45 (20–76) | **<0.01** |
| Length of ICU stay [days] (IQR) | 23 (8–34.5) | **0.01** |
| Overall complications [n (%)] | 19 (95.0) | 0.25 |
| *CDC ≥ IIIa* | 17 (85.0) | **<0.001** |
| In-hospital mortality | 8 (40.0) | **0.004** |
| Adjuvant therapy [n (%)] |  | **<0.001** |
| *Indicated* | 13 (100) |  |
| *Received* | 3 (23.0) |  |
| Overall survival [days] (CI) | 104 (56–354) | **<0.001** |

* Compared with PTP

Abbreviations: CDC, Clavien-Dindo classification; CI, 95% confidence interval; ICU, intensive care unit; IQR, interquartile range; POD, postoperative day; PTP, primary total pancreatectomy; RCP, rescue completion pancreatectomy; SOFA, Sequential Organ Failure Assessment
